# Use of a Four-Tiered Graph to Parse the Factors Leading to Phenotypic Clustering in Bacteria: A Case Study Based on Samples from the Aletsch Glacier

**DOI:** 10.1371/journal.pone.0065059

**Published:** 2013-05-31

**Authors:** Miroslav Svercel, Manuela Filippini, Nicolas Perony, Valentina Rossetti, Homayoun C. Bagheri

**Affiliations:** 1 Institute of Evolutionary Biology and Environmental Studies, University of Zurich, Zurich, Switzerland; 2 Chair of Systems Design, ETH Zurich, Zurich, Switzerland; The Centre for Research and Technology, Greece

## Abstract

An understanding of bacterial diversity and evolution in any environment requires knowledge of phenotypic diversity. In this study, the underlying factors leading to phenotypic clustering were analyzed and interpreted using a novel approach based on a four-tiered graph. Bacterial isolates were organized into equivalence classes based on their phenotypic profile. Likewise, phenotypes were organized in equivalence classes based on the bacteria that manifest them. The linking of these equivalence classes in a four-tiered graph allowed for a quick visual identification of the phenotypic measurements leading to the clustering patterns deduced from principal component analyses. For evaluation of the method, we investigated phenotypic variation in enzyme production and carbon assimilation of members of the genera *Pseudomonas* and *Serratia*, isolated from the Aletsch Glacier in Switzerland. The analysis indicates that the genera isolated produce at least six common enzymes and can exploit a wide range of carbon resources, though some specialist species within the pseudomonads were also observed. We further found that pairwise distances between enzyme profiles strongly correlate with distances based on carbon profiles. However, phenotypic distances weakly correlate with phylogenetic distances. The method developed in this study facilitates a more comprehensive understanding of phenotypic clustering than what would be deduced from principal component analysis alone.

## Introduction

For validation of phenotypic diversity and physiological functions, cultivation and characterization of single isolates is a necessary and complementary approach to community assays [1], [2], [3]. Furthermore, phenotypic analysis could serve as an aid in determining the focus of subsequent genetic studies. One possible way to obtain phenotypic profiles is through characterization tests such as Biolog PM and API ZYM. These methods have been often used for obtaining the combined metabolic profile of microbial communities [4], [5]. Strain-by-strain analysis of a community using such tests is much rarer. Since isolates can display different profiles, this procedure involves the creation of high-dimensional data, which is typically difficult to analyze. The analysis becomes more difficult as the number of strains increases, hence limiting the size of data sets that can be handled. In order to render the analysis of such data possible, statistical methods that reduce the dimensionality of the data set are often used, such as Principal Component Analysis (PCA) [6]. PCA allows to group correlated variables associated with a set of entities (here, bacterial isolates) together into factors, which are thought to reflect the latent processes from which the correlations arise. However, PCA provides no easy way to understand what these processes are, and effectively to understand where the grouping between isolates originates.

Our study represents a basic investigation into phenotypic traits and phylogenetic diversity of bacteria (members of two genera: *Pseudomonas* and *Serratia*) isolated from meltwater and mud of the Aletsch Glacier. There is a paucity of information concerning the characteristics of individual strains in extreme environments, and few studies on the phenotypic diversity of cultivable bacteria in glacial surfaces are available [7], [8].

The main objective was to explore phenotypic characteristics of tested bacterial isolates with respect to carbon utilization and enzyme profile, and to better interpret the physiological basis leading to clustering patterns. We developed and verified a novel approach based on a four-tiered graph which improves on the multivariate statistical methods traditionally used in such context.

## Materials and Methods

### Sampling Sites

In September 2008, glacial meltwater, water and mud samples were collected at two sampling sites on the Aletsch Glacier leading to a total of 8 samples (for description and details see Text S1: Supporting Materials and Methods and Figure A of Text S1). The samples were harbored in 50 mL plastic sterile tubes (VWR International AG, Dietikon) (ca 5 g –40 g of material) and kept at 4°C in the lab prior to isolation of bacteria.

### Isolation and Cultivation of Bacteria

Samples were diluted by ½ with 0.5% NaCl and vortexed for at least 1 minute. An aliquot of each sample (100 µL) was decimally diluted in 0.5% NaCl and placed on Yeast extract Tryptone (YT) and Luria-Bertani (LB) agar plates. These plates were incubated at 20°C and the colony forming units (CFU) were counted after three days. Additionally, colonies with visibly different morphologies and/or color were picked and re-inoculated on YT agar plates to check for purity. Finally each colony was grown in 150 µL YT medium in 96-well microtiter plate at 20°C for three days. From each sample, 96 isolates were selected (hence totaling 768 isolates for the study) and stored at −80°C in a 20% glycerol solution.

### DNA Extraction and PCR Amplification

DNA was extracted using the heat –cold procedure in lysis buffer [9] (for procedure details see Text S1: Supporting Materials and Methods). The amplification of 16S rRNA gene was performed using two universal primers 27F and 1492R [10] and carried out using Techne TC 512 thermal cycler (Barloworld Scientific ltd, United Kingdom). More details are given in the supporting information. The length of the PCR product was verified by electrophoresis in 0.8% agarose in TAE buffer.

Isolates for further analysis were selected using Restriction fragment length polymorphism (RFLP) (for details see Text S1: Supporting Materials and Methods) according to different RFLP pattern. PCR products of the selected ones were sequenced both in-house (ABI 3730 Sequencer) and an external facility (Microsynth, Balgach, Switzerland).

The sequences were assembled and edited manually using FinchTV 1.4.0. and Geneious Pro 3.6.2 software.

### Phylogenetic Analysis

In order to determine phylogenetic affiliation between a subset of 118 selected isolates, 16S rRNA gene sequences were subjected to a Blast search in the GenBank database using the National Center for Biotechnology Information web server (http://www.ncbi.nlm.nih.gov). To produce a dataset for phylogenetic analysis, sequences from this study were aligned with close relatives retrieved from GenBank. The multiple data alignment was carried out with ClustalW [11], and phylogenetic inference was done using the software package MEGA v4.0.2 [12]. Details on phylogenetic tree algorithms and evolutionary distance methods are given in supporting information.

### Enzymatic Activity and Carbon Source Assessment

The enzymatic activity of 33 Gram-negative isolates, three Gram-positive isolates and two independent representatives of the genera *Pseudomonas* and *Serratia* was studied using API ZYM test (Biomerieux, France) following the manufacturer’s instructions.

The carbon utilization profile of the 33 isolated Gram-negative bacteria was assessed with Biolog Phenotype Microarray™ plate 1 (PM1) according to the manufacturer’s instructions. The plates were briefly incubated at 30°C in the dark and inspected for carbon assimilation after a 1-day incubation.

### Measures of Phenotypic Distance and Statistical Analysis

The results of carbon source utilization (Biolog PM1, see supporting information Tables S1 A, B) were both codified in binary (growth/no growth) and continuous form (OD measurements). For enzymatic activities (API ZYM), the results were codified in binary form.

Pairwise distances between enzyme or carbon assimilation profiles were calculated using a normalized Hamming distance. The latter distance is defined as the number of positions at which two binary strings differ, divided by the length of the string. In this case, it corresponds to the number of enzymes or carbon, respectively, for which two strains are different, divided by the maximum possible difference. For PCA analysis of the carbon data based on Hamming distances, continuous data were transformed to binary form by applying a threshold representing 10% of the maximal OD measurement determined for the whole dataset. This threshold was chosen in order to select for unequivocal growth signals and hence to avoid false positives. Accordingly, OD values above 0.196 were codified as “1″, whereas lower values were represented by “0″.

In order to assess the correlation between datasets (enzyme or carbon assimilation), we used Pearson's correlation coefficient. Both correlation coefficient and significance tests were computed with the software MATLAB (http://www.mathworks.com/).

For the purpose of comparison to genetic relatedness, phylogenetic data were handled with the software MEGA v4.0.2, which implements the composition distance. Variation in enzyme profiles, carbon assimilation profiles, and the significance of genetic distances between the two chosen genera (*Pseudomonas* and *Serratia*) was determined using a Wilcoxon test. A prior test for normality (Kolmogorov-Smirnov test) showed that the data was not normally distributed (p<0.05).

### Four-tiered Graph

#### Motivation and principle

**Figure 1:**
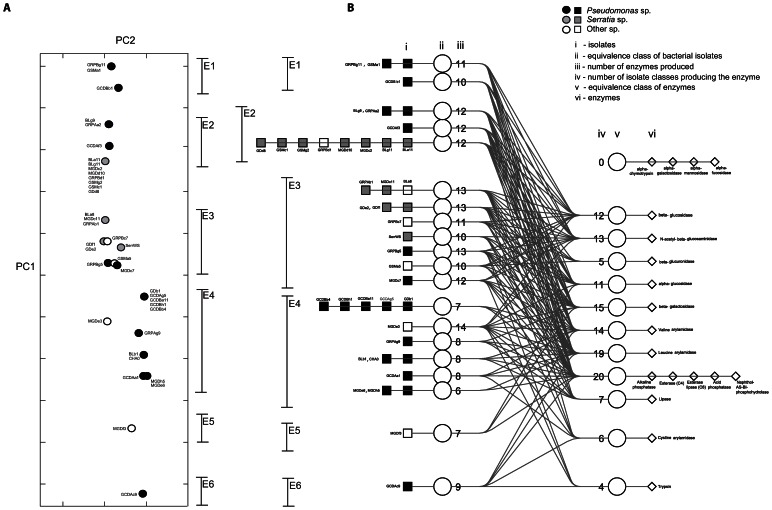
Enzyme profile analysis. (A) Principal component analysis based on Hamming distance of enzymatic profiles determined using API ZYM strips. The first two principal components explain 87% of the variance of the data. (B) Four-tiered graph linking bacteria and enzyme profiles. Links are to be followed from left to right. Bacteria showing similar enzymatic profiles (E1, E2, E3 E4, E5 and E6) group together. The number of enzymes produced by each equivalence class of bacteria and the number of bacteria classes that produce a certain enzyme are indicated at the right of the corresponding bacterial equivalence class and at the left of the corresponding enzyme equivalence class, respectively. The vertical positions of the bacterial classes correspond to their coefficient in the first principal component of Figure 1A, though vertically-overlapping classes are separated from each other by a small distance to allow for an easy reading of the graph. Distances between the non-overlapping classes are preserved.

Principal Component analysis (PCA) allows to extract from a given dataset a limited number of uncorrelated latent variables (principal components) which optimally express a large part of the data's variance in a low- (generally two- or three-) dimensional space. The correlation between two variables is often expressed by the Pearson correlation coefficient (“Pearson's r”), which provides a standardized estimate of how two variables covary. However, when one is interested in quantifying distances between vectors composed of binary variables, more straightforward metrics such as the Hamming distance can be used. For this reason, the matrices from which we computed the PCA in this study were based on a measure of similarity (expressed as one minus the normalized Hamming distance) between observations rather than the less explicit Pearson correlation coefficient (Figure 1).

Even though canonical analysis methods such as PCA are useful to reveal groups of similar observations that would otherwise remain hidden in high-dimensional data, they do not tell how these groups are constituted by the procedure. We developed a graphical method that summarizes the information given by a PCA on a network layout: it represents in a visually-clear form the relation between strain isolates grouped into phenotypic clusters, and the resources (consumed carbon sources or produced enzymes) that make up their phenotypic profile. The result is a four-tiered graph whose outermost layers comprise strain isolates on one side, and the constituents of their phenotypic profile on the other (carbon substrates/enzymes). Both strains and resources are grouped into equivalence classes, where for example two strains that consume the same carbons belong to the same bacterial equivalence class, and conversely two carbon sources that are consumed by the same strains are classified in the same resource equivalence class. A similar classification applies to enzyme production.

#### Method

**Figure 2:**
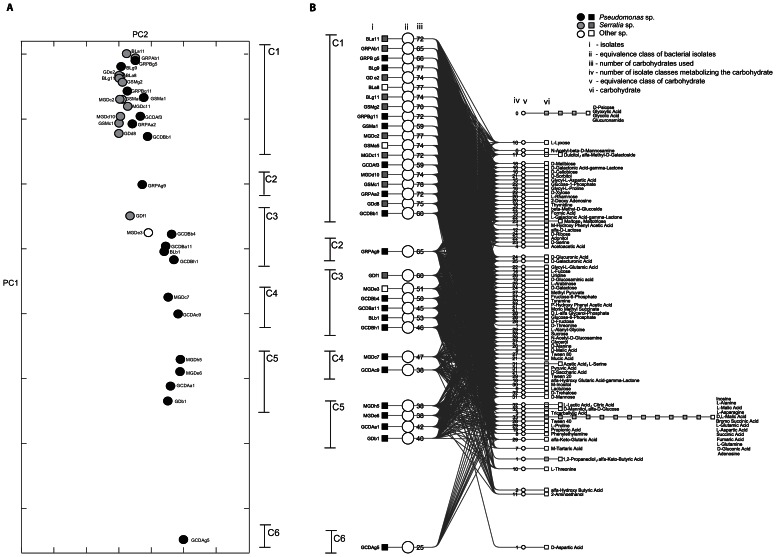
Carbon assimilation profile analysis. (A) Principal component analysis based on Hamming distance of carbon assimilation profiles measured with Biolog PM1. The first two principal components explain 84% of the variance of the data. (B) Four-tiered graph linking bacteria and carbon assimilation profiles. Bacteria showing similar carbon assimilation profiles group together (C1, C2, C3, C4, C5 and C6). This graph is constructed the same way as in Figure 1B. Here no two bacteria show identical profiles, hence they form single-member equivalence classes (each strain is linked to a unique node in the isolate equivalence class layer, second from the left). The vertical positions of the bacteria correspond to their coefficient in the first principal component of Figure 2A, though vertically-overlapping bacteria are separated from each other by a small distance. Distances between the non-overlapping classes are preserved.

In order to produce the four-tiered graph, we start by computing the equivalence classes for strain isolates on one hand, and carbon sources/enzymes on the other. Equivalence classes group together strains with the same phenotypic profile on one hand, and resources (carbon/enzyme) with the same utilization profile on the other hand. This reduces the number of items to display and improves the clarity of the visualization. As our focus is on the similarity between strains, we then organize the bacterial equivalence classes according to their coefficients in the first principal component of a PCA on their Hamming similarity matrix. As with any other PCA-based method, this gives a visual representation of the phenotypic clustering between strain isolates. When plotting the bacterial equivalence classes along a vertical line according to their coordinate on the PC1 axis, for clarity we introduce a small spacing between the classes that would otherwise overlap, whilst keeping the distances between non-overlapping classes unchanged. This allows for instant visual identification of groups of phenotypically similar strains. We then proceed to plot the resource equivalence classes on another vertical line neighbouring the one holding the bacterial classes. The resource classes are placed along this line at the barycenter of the bacterial classes to which they relate. This is completed by a simple graph layout illustrating the link between bacterial and resource classes. For example, an equivalence class of bacteria X has a link to an equivalence class of enzymes Y if all strains in class X produce the enzymes in class Y. The number of outgoing or incoming connections to each class is also shown next to it. Finally, each class is linked to the sequence of bacterial isolates or carbon sources/enzymes that it includes (more information in Figures 1 and 2 and their caption).

### Algorithm

**Figure 3:**
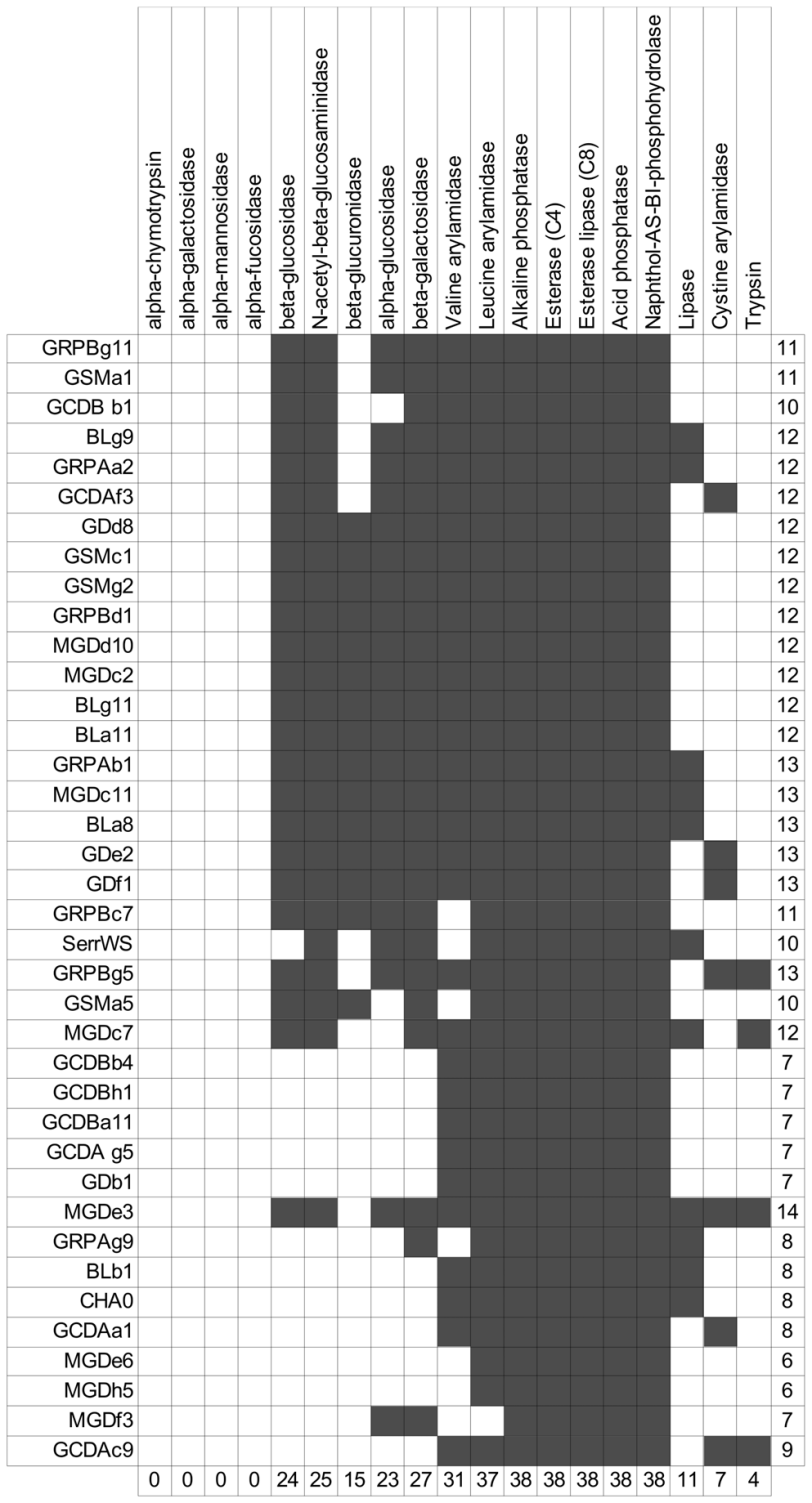
Overview of enzymatic profiles for all strains. Profile of the strains presented in Figure 1 (four-tiered graph), listing the activity of the different enzymes (API ZYM strip) in each isolate or equivalent class of bacterial isolates. The ordering of the strains and the enzymes in the table was modified to match the graph in Figure 1.

**Figure 4:**
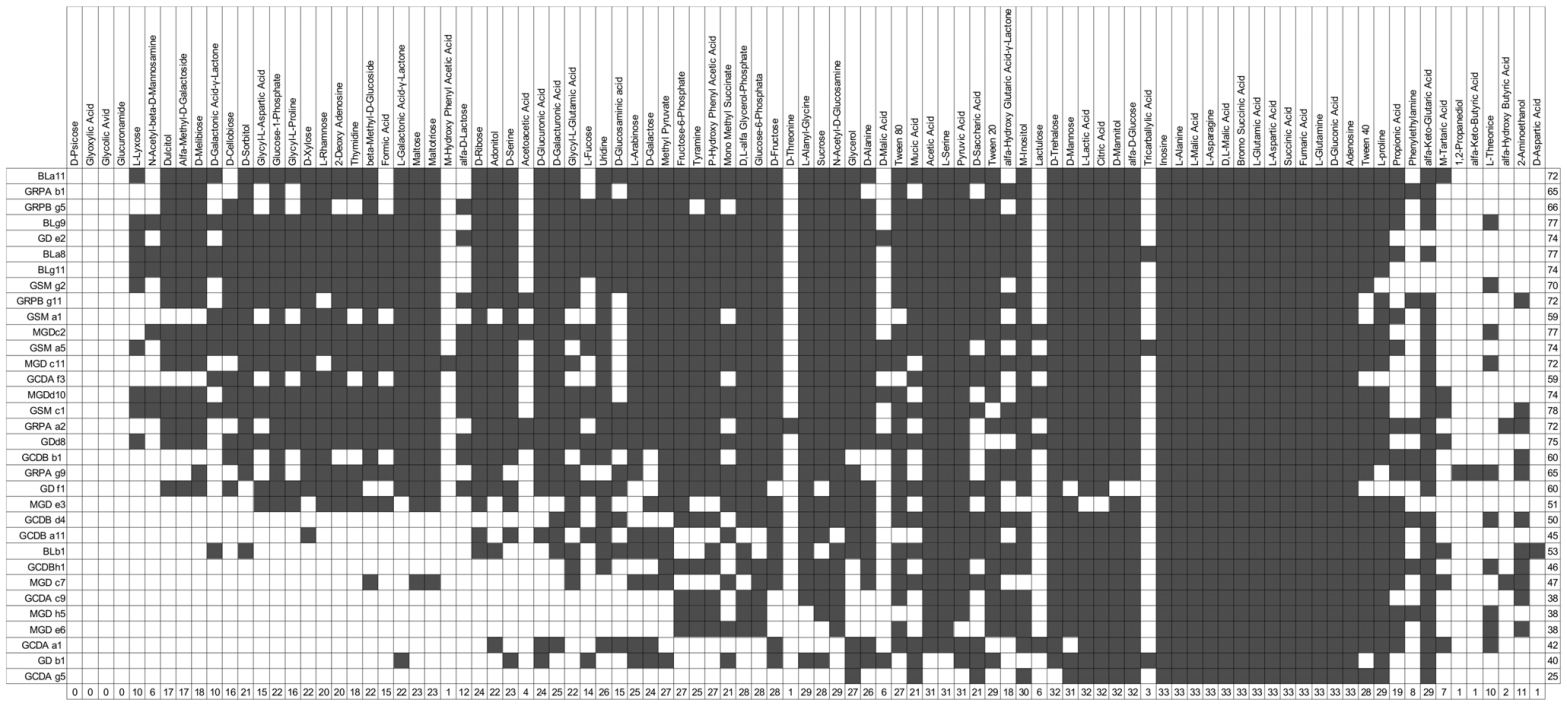
Overview of carbon assimilation profiles for all strains. Profile of the strains presented in Figure 2 (four-tiered graph), listing the carbon substrates (BIOLOG 1) used by each isolate or equivalent class of bacterial isolates. The ordering of strains and the carbon substrates in the table was modified to match the graph in Figure 2.

The algorithm is written in MATLAB code and available to download, along with the data files (illustrated in Figures 3 and 4) used for this article, in the Supporting information section (the code has been commented and should be easy to follow for regular MATLAB users; we have also provided a sample data file that can be used to generate the graph of Fig. 4A). The procedure is the following: first, names of strains and resources (carbon sources/enzymes) and their binary relation are extracted from a plain text file containing comma-separated values. In a second step, strains and resources are grouped into equivalence classes and their contents and profiles listed. Then, all strains, strain equivalence classes, resource equivalence classes and resources are ordered in a directed association matrix which is used by the layout algorithm. Finally, the coordinates of all strains/classes/resources, or nodes of the graph, are calculated as per the method described above and the output is written to the open source GraphML file format (http://graphml.graphdrawing.org/) for maximum compatibility. The result can be visualized and manipulated using a number of tools supporting the GraphML format, including the free editor yEd (http://www.yworks.com/en/products_yed_about.html), which we used to produce the figures of the present article. Whereas the figures presented here are static, the relations between strains, classes and resources can be visualized from the GraphML file in a dynamic fashion, allowing for example to highlight the relation to and from an equivalence class of strains or resources by clicking on it.

### Nucleotide Sequence Accession Numbers

The 36 nucleotide sequences of partial 16S rRNA genes of strains tested phenotypically in this study have been deposited in GenBank with accession numbers GU939679 to GU939714.

## Results

From a total of 768 single isolates selected - from different environmental subsites (96 isolates per subsite, Figure Ac of Text S1), 118 isolates were chosen according to different RFLP profiles for sequencing. Most (85%) of the sequenced isolates belong to genera *Pseudomonas* and *Serratia* (Figure Ac of Text S1) and between the two genera, higher phenotypic variation was observed in *Pseudomonas* than in *Serratia* (supporting Figure B of Text S1). The phylogenetic relationship of the 33 Gram-negative isolates used to test our method can be seen in supporting Figure C of Text S1.

### Enzyme Activity and Carbon Assimilation

Enzyme activity data of several samples, in our case different isolates, are generally analyzed by PCA. The first and second principal components of a PCA analysis of the relationship between bacteria and their ability to produce active enzymes explained together 87% of the variance (80% and 7%, respectively) in the data (Figure 1A). From this analysis, researchers gain exclusively information on the clustering pattern. In this respect we observed that *Pseudomonas* spp. and *Serratia* spp. occupy distinct, though sometimes overlapping, ecological niches or ecotypes (Figure 1A). More information can be gained out of these kinds of datasets, for example why bacteria group together and/or which variable is present/absent in different classes.

In order to understand the underlying phenotypic profiles that lead to the clusters produced by PCA, bacterial and enzymatic equivalence classes were therefore linked together in a four-tiered graph. This new approach allow for a clearer overview of the differences and similarities between enzyme profiles of different bacterial groups (Figures 1B and 3). Additionally, functional classes can be derived from this analysis. In group E1, there are two bacterial equivalence classes belonging to the genus *Pseudomonas*. In group E2, there are two bacterial classes with *Pseudomonas* and one class with *Serratia* and *Bacillus*. In group E3, there are seven classes composed of a mix of two *Pseudomonas* spp., two *Yersinia* spp., one *Viridibacillus* sp. and five *Serratia* spp. In the fourth PCA group (E4), there are five homogenous classes with *Pseudomonas* sp. and one with *Aeromonas* sp. In the fifth (E5) and sixth (E6) group, there is one representative of *Exiguobacterium* sp. and one of *Pseudomonas* sp., respectively. It can be observed that this approach allows us to discover the causes of the grouping. For example, the E4 group (Figure 1B) composed of *Pseudomonas* sp. isolates exhibited a lower number of enzymes, and in comparison with the other groups, did not produce β-glucuronidase, α-glucosidase, β-glucosidase, N-acetyl-β-glucosaminidase, trypsin, and β-galactosidase (with one exception for the latter: GRPAg9). Groups E1, E2 and E3 are the groups that on average produced more enzymes than the other groups. Additionally, five enzymes were produced by all strains: alkaline phosphatase, esterase (C4), esterase lipase (C8), acid phosphatase, and naphthol-AS-BI-phosphohydrolase. Furthermore, leucine arylamidase was produced by 35 out of 36 strains. The enzyme activity of alpha-chymotrypsin, alpha-galactosidase, alpha-mannosidase and alpha-fucosidase could not be detected for any strain. 25 isolates (69% of strains) produced more than eight enzymes, whereas the remaining 11 bacteria (31%) produced eight or less enzymes.

The method presented here can also be applied to datasets with more variables. Here, we report the example of carbon assimilation profiles done with BIOLOG plates, where 95 variables were present. Similarly to the enzymes, the PCA analyses of the carbon utilization profiles indicated that the first two principal components could explain 84% of variance in the data (74% PC1 and 8% PC2). Additionally, six functional groups could be distinguished (Figure 2A). Group C1 was composed by *Pseudomonas* spp. and almost all *Serratia* spp. isolates. The other groups (C2, C3, C4, C5 and C6) were mainly composed of *Pseudomonas* spp. For this dataset too, bacteria and carbon assimilation profiles were linked in a four-tiered graph in order to understand the underlying phenotypic profiles that led to the PCA clusters (Figure 2B). Differently to the enzymes, all bacterial equivalence classes had only one member, indicating that all profiles differed by at least one carbon source. An increase in the number of variables studied will create this outcome in most cases: as no two strains present then the same profile, the graph includes many line crossings and becomes hard to read. This is an argument in favor of limiting the number of variables used to characterize strain isolates with a four-tiered graph. However, opening the graph with adequate software (e.g. yEd) makes the interpretation easier since each profile can be highlighted separately, which improves the readability of the figure when compared to a static layout (such as those presented in this article).

Additionally, Figure 2 shows that (i) four substrates were not used by any of the bacteria: glucuronamide, D-psicose, glyoxylic acid and glycolic acid. (ii) For a subset of 13 of the 95 carbon sources, all isolates were capable of metabolizing them after 24 h. These compounds (supporting Table A of Text S1) were predominantly amino acids (38.5%) and carboxylic acids (38.5%). Seven substrates (supporting Table B of Text S1), which were mostly comprised of carboxylic acids, were used by less than 10% of the bacteria selected. (iii) The total number of substrates used range from a minimum of 25 (26% of total number of substrates) metabolized by pseudomonad (GCDAg5), to a maximum of 78 substrates (82% of the total number of substrates) by the *Serratia* sp. GSMc1 isolate. (iv) According to the number of C-resources metabolized, the isolated bacteria can be divided into mainly six groups: one group (C1) with 22 bacteria using between 60 and 78 substrates, the second group (C2) with one bacterium using 65 substrates, the third group (C3) with six bacteria using on average 50 substrates, the forth group (C4) with two bacteria using on average 42 substrates, the fifth group (C5) with four bacteria using 39 substrates on average, and the sixth group (C6) with only one member (GCDAg5) metabolizing 25 substrates. (v) Among the *Serratia* spp., the average number of resources metabolized was 72, a value higher than the average of 52 for *Pseudomonas* spp. This latter group exhibits a broader range of values (25–77).

### Correlation between Enzyme Activity, Carbon Source Usage and Phylogeny

An evaluation of a linear relationship between variables was made using Pearson's correlation coefficient. Enzyme distance strongly correlated with the carbon distance (corr. coef. ρ = 0.75, p<0.001; supporting Figure Da of Text S1) and both in turn weakly correlated to the phylogenetic distance (corr. coef. ρ ranging from 0.30 to 0.35, p<0.001; supporting Figure Db and c of Text S1).

## Discussion

In this study, we investigated phenotypic diversification of two genera obtained from the same environment by means of a novel graphical tool, a four-tiered graph of interactions between bacterial isolates and phenotypic characteristics (variables). The method is proposed as a complementary tool to classical PCA analysis, as it complements traditional methods by adding information as to “why” isolates cluster together. As the method is based on concepts from graph theory, it is intuitive, easy to use, and can scale up to handle large datasets if necessary. Beside the static output file (typically a PDF document), a dynamical visualization with dedicated software (e.g. yEd) helps in the interpretation of data, especially if a large data set is analyzed. A node corresponding to an equivalence class (for example of isolates) can be graphically highlighted, making it possible to follow its phenotypic profile and understand the clustering observed. In comparison to results given by tables, the outcome of the four-tiered graph is clearer and offers a bilateral reading giving complementary information. In our case, looking at the left-hand side (strain isolates), information on group composition can be obtained, indicating for example if isolates from the same genera are clustered together or mixed with other genera (possible explanation: convergent evolution or/and gene exchange). A look at the resource side (right-hand side) indicates which variables (in our case carbons or enzymes) are commonly used, which ones are not and which ones are responsible for the clustering.

Depending on the research question, it might be helpful to select only for relevant variables with respect to the studied environments. Not only will this reduce the complexity of the graph, but it also will improve the outcome of the method by getting rid of irrelevant variables acting as a noise source on the clustering patterns, thus allowing for a better interpretation of the results. Finally, the source code from our implementation of the method is available to download, giving other researchers the possibility to use and adapt it according to their needs and expectations.

It should be noted that use can be made of the four-tiered graph in any context where generic entities are characterized by a binary profile. Applications of the technique thus reach well beyond microbiology, as one could think of using it to visually interpret transformations of sequencing data (where organisms are characterized by the presence or absence of specific genes), or even in a social network context, where clusters of similarly-minded individuals can be compared by their common preference for a given network feature (e.g., Facebook friends and page likes). The introduction of our method in this paper is a step toward the generalization of graph visualization techniques in the exploration of complex data sets.

In the next paragraphs we will discuss the results of the Aletsch isolates data set used to validate the four-tiered graph method. This provides an example of discussion points that researchers can address using the new tool. The genera cultivated and identified from the Aletsch sampling sites are known to be widely spread in cold environments, though their presence and abundance varies according to the sampling sites and the detection methods used. Gram-negative bacteria such as *Pseudomonas*, *Serratia*, *Yersinia* and/or *Janthinobacterium* are found to be broadly predominant in glaciers in the European Alps [13], [14], in Asia [15], [16], [17] and in some Antarctic or Arctic glaciers [18], [19], [20]. Concerning other studies, high variation in microbial biomass and community structure have been revealed by comparison of geographically distinct glaciers worldwide [15], [18], [19], [21], [22], [23], [24], [25]. This variation is highly influenced by climatic and environmental factors, including geographic location [26], [27], wind direction and speed, light intensity, precipitation (snow and rain), and availability of nutrients and liquid water [21], [28], [29]. Changes in bacterial assemblages were also investigated between and within different habitats (snow, slush, and lake water) [30]. Additionally, surveys concerning microbial communities on a broad geographical scale indicate that microbes may be divided into ubiquitous and endemic groups; the former are able to establish under a broad range of environmental conditions, the latter are more specifically adapted to the unique characteristics of the site [22].

Several interesting observations arose from our study. Bacteria isolated from the Aletsch glacier could be classified into several categories, whether using enzymatic activity (API ZYM strip) or carbon assimilation (Biolog PM1) tests. With some exceptions, the different clusters identified in both API ZYM and Biolog tests are composed of mostly the same members. Accordingly, a significant positive correlation for distances between enzyme profiles and carbon assimilation profiles (supporting Figure Da of Text S1, corr. coef. ρ = 0.75, p<0.001) indicated that clustering based on enzymes or carbon sources will be quite similar.

An examination of the clusters observed in the phenotypic analyses indicates that there are groups within which members of different genera have similar phenotypic profiles such as E2 and E3 for the enzyme profiles (Figure 1), and group C1 for the carbon source profiles (Figure 2). A possible explanation could be convergent evolution of separate lineages. Gene exchange between the two groups is also a possibility. Nonetheless it is noteworthy that we did not find a case where *Pseudomonas* and *Serratia* were in the same equivalence class. A comparison between genomes from representative clusters could potentially resolve some of the questions with regard to the origin of these convergent profiles.

The enzymes (alkaline phosphatase, esterase (C4), esterase lipase (C8), acid phosphatase, naphthol-AS-BI-phosphohydrolase and leucine arylamidase) produced by all *Pseudomonas* and *Serratia* are responsible for the hydrolysis of molecules that can subsequently be transported into the cell. Additionally, the presence of these enzymes was reported in several other studies such as a microbial community on a Glacier [31], in cryoconites in Antarctica [32], in marine beach sediments [33], and at the air-water interface of an estuarine lake [34]. This overall presence suggests that these enzymes are potentially core enzymes and essential for the survival of the organisms.

The C-assimilation results demonstrated that the isolates were capable of exploiting several different types of carbohydrates, amino acids, and carboxylic acids, suggesting the presence of metabolically diverse heterotrophic bacteria on the Aletsch glacier. According to the classification by Liu et al [8], where bacteria isolated from Mount Everest were classified as versatile in utilization of carbon substrates, the isolated bacteria in our study tend to be generalists rather than specialists relying on very few substrates (with the exception of *Pseudomonas* GCDAg5, which could only use 25 of the carbon sources). This is different to the results of Foreman et al. [32], who observed that microbes within cryoconites were capable of metabolizing a maximum of 17 substrates, supporting the idea of specialized bacteria. Given that no two carbon assimilation profiles were identical, results suggested that each isolate could in principle occupy a unique niche and therefore not necessarily always compete with others for the same critical resource. This diversification in terms of assimilation could be the result of the principle of competitive exclusion as suggested by Gause [35]. Nonetheless, it should be noted that our results are based on a predetermined sampling of metabolites. However, the assay may not necessarily reflect the real spectrum and diversity of resources the bacteria are exposed to in the environment. Moreover, our isolates may not represent active bacteria or could be just dormant *in situ*. Additionally, as we already mentioned in the introduction, there is a paucity of information concerning the characteristics of individual strains in extreme environments, and only few studies on the phenotypic diversity of cultivable bacteria in glacial surfaces are available [7], [8]; the tested cryophilic genera tested in these studies were different to ours. Therefore, expressing our results in the context of previously reported studies of glacial microbiology may be more speculative than sound. Concerning the Swiss Alps, previously unexplored niches for microbial life have been discovered beneath glaciers [36]. Knowledge of this environment is also derived from studies on bacterial succession in glacial forefield soil [37], [38], [39]. However not much information on cultivable bacteria inhabiting meltwater derived from Swiss Glaciers are available. The aforementioned studies have been able to recover viable and diverse cultivable bacteria having different survival strategies. Up to now, not much is known about physiological functions such as utilization of carbon sources and enzymatic production of glacial bacteria, which could represent a key to their survival and growth ability in cold environments such as glaciers, snow and ice.

Weak positive correlations between phenotypic signals (enzymatic activity and carbon assimilation) and phylogeny based on 16S rRNA gene sequences were observed. This weak correlation can be explained by the fact that *Serratia* spp. isolates have several phenotypic similarities to *Pseudomonas* and hence overlap in their profile (possibly due to convergent evolution). Moreover, *Pseudomonas* spp. isolates are very variable in terms of their phenotypic profiles; in our analysis we can distinguish between two main groups which are far from each other. Additionally, phylogenetically similar *Serratia* isolates (based on the 16S rRNA gene) can still exhibit some variation in enzyme activity and carbon assimilation profiles (e.g., BLa11, MGDc11 and GDe2). Likewise, a pattern of phenotypic diversification was also observed in a set of phylogenetically similar *Pseudomonas* strains (e.g., GCDAc9, GCDBb1, GCDBb4, GRPAg9 and MGDh5). These results could indicate phenotypic plasticity and/or higher genetic diversity in loci other than the 16S rRNA gene. Furthermore, isolates with the same enzymatic profile can have different carbon profiles and belong to different species (e.g., GCDBb4, GCDBh1 and GDb1). The higher variation in carbon profile than in enzymes is primarily due to the higher number of carbon sources tested (95 C-substrates) in comparison to the enzymes (19 enzymes).

Using members of two genera isolated from the Aletsch Glacier as a case study, we tested a method we developed for interpretation of phenotypic clustering patterns. The analysis provides a heuristic tool for understanding the similarities and differences between the phenotypic profiles of different isolates. An investigation of the relation between phenotype and phylogenetic signals in this study suggests that bacteria that are phylogenetically distant can exhibit similar phenotypic profiles when isolated from the same environment. Hence physiological characterization does not necessarily help distinguish between different genera of bacteria isolated from the same environment. Likewise, it also means that species diversity in an environment does not necessarily imply phenotypic diversification (see also [2]). These are important considerations for bacterial identification schemes based solely on phenotypic profiles. The same considerations also apply to ecological and evolutionary studies based solely on sequence-based species diversity profiles.

## Supporting Information

Text S1 (PDF)
