# Correction: Use of a Four-Tiered Graph to Parse the Factors Leading to Phenotypic Clustering in Bacteria: A Case Study Based on Samples from the Aletsch Glacier

**DOI:** 10.1371/annotation/3f02ad8b-6dac-4460-a969-90b3f5eb1306

**Published:** 2013-06-18

**Authors:** Miroslav Svercel, Manuela Filippini, Nicolas Perony, Valentina Rossetti, Homayoun C. Bagheri

There is a Supporting Information file that is missing from the article. The following is the legend and link to the missing file:

File S1. MatLAB code. (ZIP) 
