# Supplementary material for: Use of a Four-Tiered Graph to Parse the Factors Leading to Phenotypic Clustering in Bacteria: A Case Study Based on Samples from the Aletsch Glacier
Source: PLoS One. 2013 May 31;8(5):e65059. doi: 10.1371/journal.pone.0065059 (PMC3669021; doi:10.1371/journal.pone.0065059)
Supplement: Text S1 — (PDF) [file pone.0065059.s001.pdf]

Text S1

Use of a four-tiered graph to parse the factors leading to phenotypic clustering in bacteria: a case study based on samples from the Aletsch Glacier

Miroslav Svercel[1*§], Manuela Filippini[1*], Nicolas Perony[2*], Valentina Rossetti[1], Homayoun C. Bagheri[1§]

1 Institute of Evolutionary Biology and Environmental Studies, University of Zurich, Zurich, Switzerland, **2** Chair of Systems Design, ETH Zurich, Zurich, Switzerland

[*] These three authors contributed equally to this work

[§] Corresponding authors, E-mails: miro.svercel@gmail.com

bagheri@ieu.uzh.ch

Text S1: Supporting Materials and Methods

Sampling sites

In sampling site A, located at the edge of the Aletsch glacier, six different subsites were chosen for sampling (supporting Figure A of Text S1): "Blue lagoon" (BL) – small water pond situated 15-20 m east of the glacier; "Glacier drip" (GD) – drip water from glacier; "Glacier cave drip" (GCD) – water drip from the roof of a glacier cave (two samples taken labeled as GCDA, GCDB); "Glacier surface mud" (GSM) – mud particles covering the top part of glacier, "Mud under Glacier Drip" (MGD) – small mud ponds collecting under dripping water from edges of the glacier. At sampling site B, on a hilly ridge to the south of the glacier, two samples were collected and labeled "Grass reactor pond A and B" (GRPA, GRPB) – small ponds (approx. 16 m^2) situated outside the glacial body (about 100 m) at an altitude of 2175 m above sea level, with floating grass debris and brownish water.

DNA extraction and PCR amplification

To extract DNA, a 30 µL aliquot of bacterial suspension from each cultivated isolate was transferred into 100 µL lysis buffer (50 mM KCl; 0.1% Tween 20; 10 mM Tris-HC; pH 8.3) [9] in an ABgene® 96-well PCR plate (Thermo Fisher Scientific Inc., Waltham, USA). The suspension was centrifuge at 2880 × *g* for 5 min and incubated for 10 min at 99°C. The heat-lysed suspension was frozen at -20°C for 30 min. When thawed, 7 µL of the supernatant was taken for PCR. Amplification of 16S rRNA genes was performed using universal primers for bacteria 27F and 1492R [10] and carried out in 30 µL reaction mixtures containing 7 µL of lysed bacterial suspension, 12 µL of RNase-free water, 12 µL of TopTaq Master Mix (Qiagen, Hombrechtikon, Switzerland) and 1.5 µL of each primer (0.40 µM). The PCR was done using

the Techne TC 512 thermal cycler (Barloworld Scientific ltd, United Kingdom) with the following program: 5 min at 95°C, 37 cycles of denaturation at 95°C for 45 s, annealing at 58°C for 40 s and extension at 72°C for 120 s. The thermo cycling condition was concluded with a final extension at 72°C for 10 min.

Restriction fragment length polymorphism and 16S rRNA gene sequencing of selected bacteria

The PCR amplicons were digested with four fastDigest® restriction enzymes *Hae*III, *Hinf*I, *Msp*I and *Taq*I (LabForce AG, Nunningen, Switzerland) according to the supplier's instructions. Restriction fragments were separated by electrophoresis in ethidium bromide stained 2% agarose gels. Isolates were selected for further investigation on the basis of different RFLP pattern profiles using the combination of above mentioned restriction enzymes. Prior to sequencing, PCR products were purified using the QuickStep^{TM} 2 96-well PCR purification kit (EdgeBio, Gaithersburg, USA). The sequencing was performed in-house (ABI 3730 Sequencer) and confirmed by re-sequencing at an external facility (Microsynth, Balgach, Switzerland).

Phylogenetic analysis

Phylogenetic trees were constructed by using Neighbor-Joining (NJ), minimal evolution (ME), maximum parsimony (MP) and maximum likelihood (ML) algorithms using MEGA software package. Evolutionary distances were computed using Kimura-2 model and Maximum Composite Likelihood methods. Maximum likelihood was performed using Garli [S1]. The TIM3 +G model (Transitional Model3, +G, gamma correction) was determined as the appropriate model of molecular evolution for our data by using jModeltest 0.1.1 [S2]. Analyses were performed using bootstrap values of 1000. The phylogenetic analysis was performed based

TABLE A. Carbon substrates used by all tested bacteria

Amino Acids	Carboxylic Acids	Miscellaneous
L-Aspartic Acid	Succinic Acid	Adenosine
L-Glutamic Acid	D-Gluconic Acid	Inosine
L-Asparagine	Fumaric Acid	Bromo Succinic Acid
L-Glutamine	D,L-Malic Acid	
L-Alanine	L-Malic Acid	

TABLE B. Carbon substrates used by less than 10% of the tested bacteria

Carbohydrates	Amino Acids	Carboxylic Acids
1,2 propanediol	D-Aspartic Acid	Alfa-hydroxy butyric acid
	D-Threonine	Alfa-keto-butyric acid
		Tricarballylic Acid
		M-Hydroxy Phenyl Acetic Acid

Classification was done according to Preston-Mafham et al. [S7].

Figure Legends of Figures in Text S1

Figure A of Text S1. Map of the sampling sites.

(a) Aerial view of the Aletsch Glacier (Canton Valais, Switzerland) showing the two sampling sites investigated in this study. Modified from International Space Station Imagery (ISS013-E-77377, 5 Sep. 2006). (b) Topographic map of the sampling region showing the location of the two sampling sites. Created using OpenStreetMap and Marble Desktop Globe version 0.8.5. (c) Sampling site codes and numbers of isolates tested.

Figure B of Text S1. Box plots of genetic variance.

Box plot of genetic variance data based on the whole data set of 16S rRNA gene sequences of *Pseudomonas* and *Serratia* genera. For box plots, the central mark is the median, and the edges of the box are the 25th (Q1) and 75th (Q3) percentiles. The maximum whisker length is determined by w = 1.5. Whiskers extend to the most extreme data points falling within the range [Q1-w*(Q3-Q1), Q3+w*(Q3- Q1)]. Data points outside this range are plotted individually as crosses. Significance of the difference in medians between the two genera was tested with the Wilcoxon test.

Figure C of Text S1. Neighbor-joining phylogenetic tree of 16S rRNA sequences of sampled γ-*Proteobacteria* representatives and their closest relatives.

Clade A represents the *Pseudomonas* genus whereas clade B is a subset of the *Enterobacteriaceae*. Codes in bold indicate strains isolated in the present study. Bootstrap values >50% are shown at the nodes as a percentage of 1000 replicates. Scale bar represents number of changes per nucleotide position. Accession numbers of 16S rRNA gene sequences are given in brackets. The tree was rooted using *Aquiflex pyrophilus* as the outgroup.

Many of our Gram-negative isolates were pseudomonads (clade A), and members of three subclades (A1, A2 and A3) matched with psychrophilic bacteria obtained from Arctic, Antarctic and glacial environments [S3,S4]. *Serratia* strains GDd8 and MGDd10 matched to psychrotrophic *S. proteamaculans* from food [S5]. A large group of *Serratia* was affiliated to strain C1 (subclade B2), which was found in the gut of the Tsetse fly [S6].

Figure D of Text S1. Scatter plot analysis.

(a) Scatter plot of pairwise distances between enzymes profiles (API ZYM) and carbon assimilation profiles (Biolog PM1), both based on normalized Hamming distance. (b) Scatter plot of pairwise distances between enzymes profiles and phylogenetic distance. (c) Scatter plot of pairwise distances between carbon assimilation profiles and phylogenetic distance (c). Coordinate of each point represents a variable pair measured for a given bacterium. The Pearson correlation coefficient was applied in order to establish correlation between datasets. The significance of the latter coefficient was tested through a t-test.

Figure A.

a

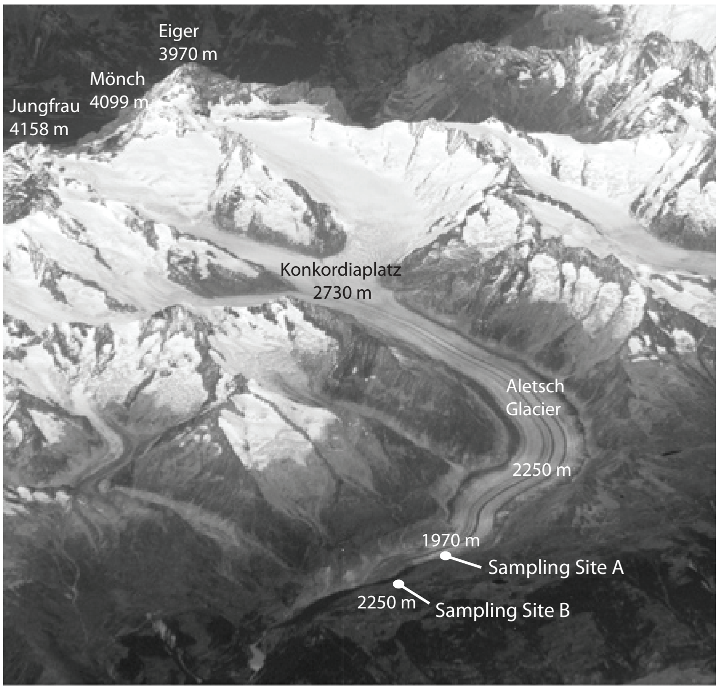

b

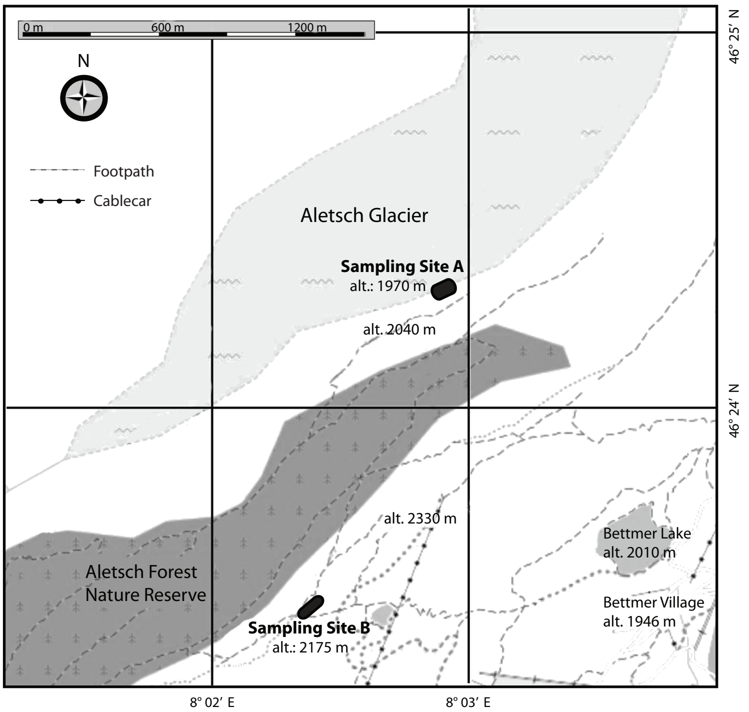

c

Site	Subsite	Sample code	Isolates for RFLP	Sequenced isolates	Phenotypically tested isolates
A	Blue Lagoon	BL	96	12 (P-7; S-3)[a]	5
"	Glacier Drip	GD	96	8 (P-4; S-4)	4
"	Glacier Cave Drip A	GCDA	96	16 (P-10, S-5)	4
"	Glacier Cave Drip B	GCDB	96	12 (P-11, S-0)	4
"	Glacier Surface Mud	GSM	96	5 (P-1; S-2)	4
"	Mud under Glacier Drip	MGD	96	41 (P-20; S-12)	8 (7)[b]
B	Grass Reaction Pond A	GRPA	96	14 (P-3; S-11)	3
"	Grass Reaction Pond B	GRPB	96	10 (P-3, S-4)	4 (2)
Total isolates			768	118 [c](P-59, S-41)	36 (33)

[a] Numbers of *Pseudomonas* (P) and *Serratia* (S)

[b] Numbers of bacteria tested in Biolog (Gram-positive bacteria were not tested)

[c] Beside P and S, additional genera (belonging to *Yersinia* sp., *Aeromonas* sp., *Janthinobacterium* sp. and to Gram-positive: *Bacillus* sp., *Viridibacillus* sp. and *Exiguobacterium* sp.) were isolated from samples

Figure B.

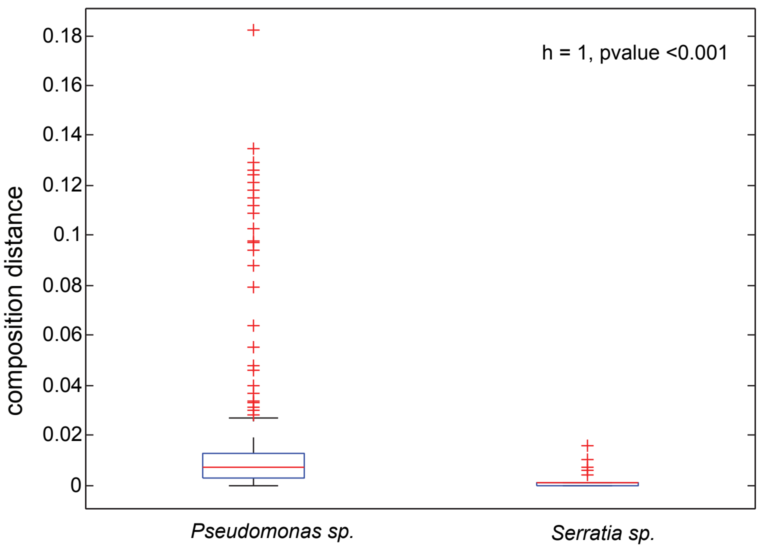
h = 1, pvalue <0.001
composition distance
0.18
0.16
0.14
0.12
0.1
0.08
0.06
0.04
0.02
0
Pseudomonas sp.
Serratia sp.

Figure C.

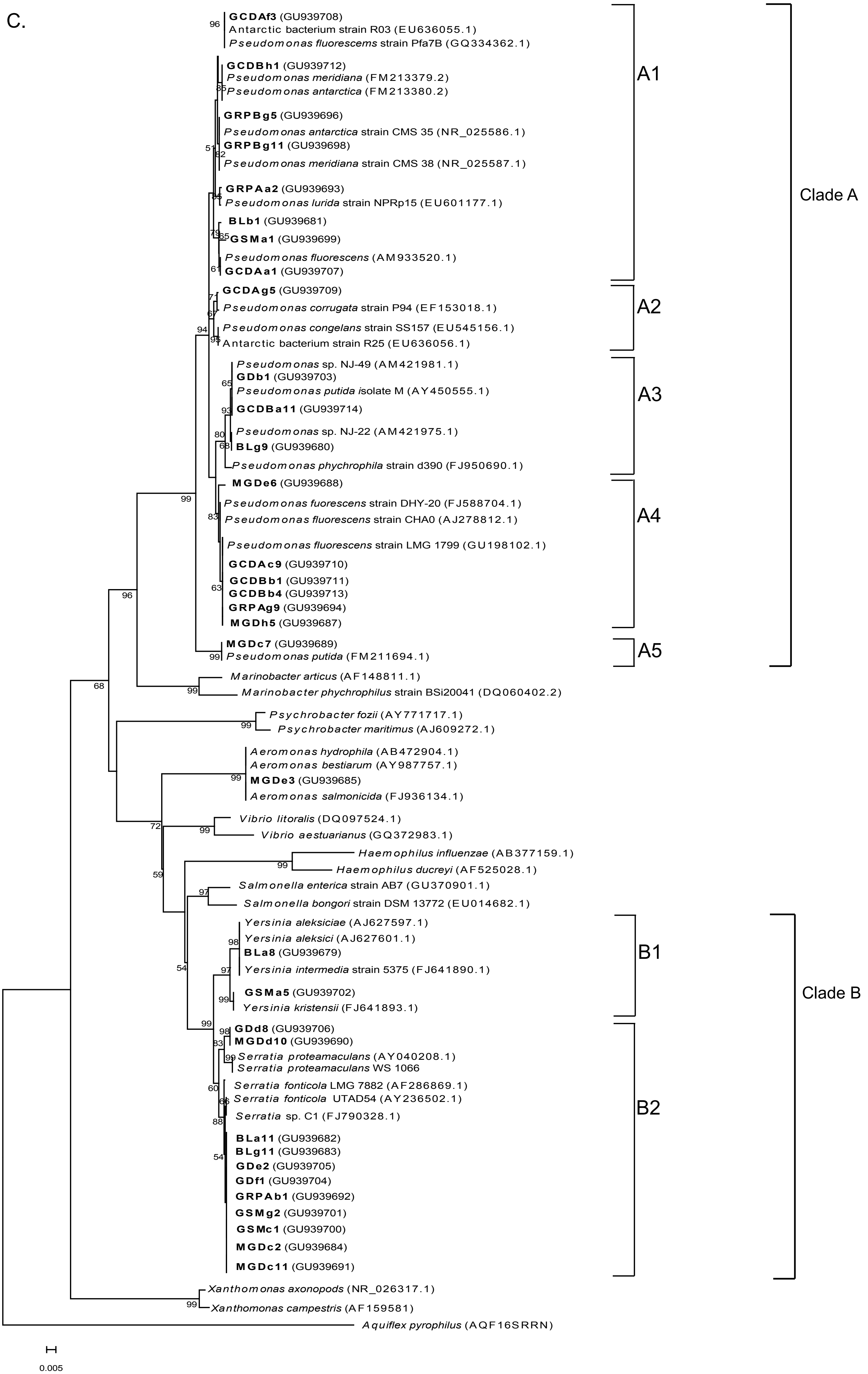
GCDAf3 (GU939708)
Antarctic bacterium strain R03 (EU636055.1)
Pseudomonas fluorescems strain Pfa7B (GQ334362.1)
GCDBh1 (GU939712)
Pseudomonas meridiana (FM213379.2)
Pseudomonas antarctica (FM213380.2)
GRPBg5 (GU939696)
Pseudomonas antarctica strain CMS 35 (NR_025586.1)
GRPBg11 (GU939698)
Pseudomonas meridiana strain CMS 38 (NR_025587.1)
GRPAa2 (GU939693)
Pseudomonas lurida strain NPRp15 (EU601177.1)
BLb1 (GU939681)
GSMa1 (GU939699)
Pseudomonas fluorescens (AM933520.1)
GCDAa1 (GU939707)
GCDAg5 (GU939709)
Pseudomonas corrugata strain P94 (EF153018.1)
Pseudomonas congelans strain SS157 (EU545156.1)
Antarctic bacterium strain R25 (EU636056.1)
Pseudomonas sp. NJ-49 (AM421981.1)
GDb1 (GU939703)
Pseudomonas putida isolate M (AY450555.1)
GCDBa11 (GU939714)
Pseudomonas sp. NJ-22 (AM421975.1)
BLg9 (GU939680)
Pseudomonas phychrophila strain d390 (FJ950690.1)
MGDe6 (GU939688)
Pseudomonas fuorescens strain DHY-20 (FJ588704.1)
Pseudomonas fluorescens strain CHA0 (AJ278812.1)
Pseudomonas fluorescens strain LMG 1799 (GU198102.1)
GCDAc9 (GU939710)
GCDBb1 (GU939711)
GCDBb4 (GU939713)
GRPAg9 (GU939694)
MGDh5 (GU939687)
MGDc7 (GU939689)
Pseudomonas putida (FM211694.1)
Marinobacter articus (AF148811.1)
Marinobacter phychrophilus strain BSi20041 (DQ060402.2)
Psychrobacter fozii (AY771717.1)
Psychrobacter maritimus (AJ609272.1)
Aeromonas hydrophila (AB472904.1)
Aeromonas bestiarum (AY987757.1)
MGDe3 (GU939685)
Aeromonas salmonicida (FJ936134.1)
Vibrio litoralis (DQ097524.1)
Vibrio aestuarianus (GQ372983.1)
Haemophilus influenzae (AB377159.1)
Haemophilus ducreyi (AF525028.1)
Salmonella enterica strain AB7 (GU370901.1)
Salmonella bongori strain DSM 13772 (EU014682.1)
Yersinia aleksiciae (AJ627597.1)
Yersinia aleksici (AJ627601.1)
BLa8 (GU939679)
Yersinia intermedia strain 5375 (FJ641890.1)
GSMa5 (GU939702)
Yersinia kristensii (FJ641893.1)
GDd8 (GU939706)
MGDd10 (GU939690)
Serratia proteamaculans (AY040208.1)
Serratia proteamaculans WS 1066
Serratia fonticola LMG 7882 (AF286869.1)
Serratia fonticola UTAD54 (AY236502.1)
Serratia sp. C1 (FJ790328.1)
BLa11 (GU939682)
BLg11 (GU939683)
GDe2 (GU939705)
GDf1 (GU939704)
GRPAb1 (GU939692)
GSMg2 (GU939701)
GSMc1 (GU939700)
MGDc2 (GU939684)
MGDc11 (GU939691)
Xanthomonas axonopods (NR_026317.1)
Xanthomonas campestris (AF159581)
Aquiflex pyrophilus (AQF16SRRN)
A1
A2
A3
A4
A5
Clade A
B1
B2
Clade B
0.005

Figure D.

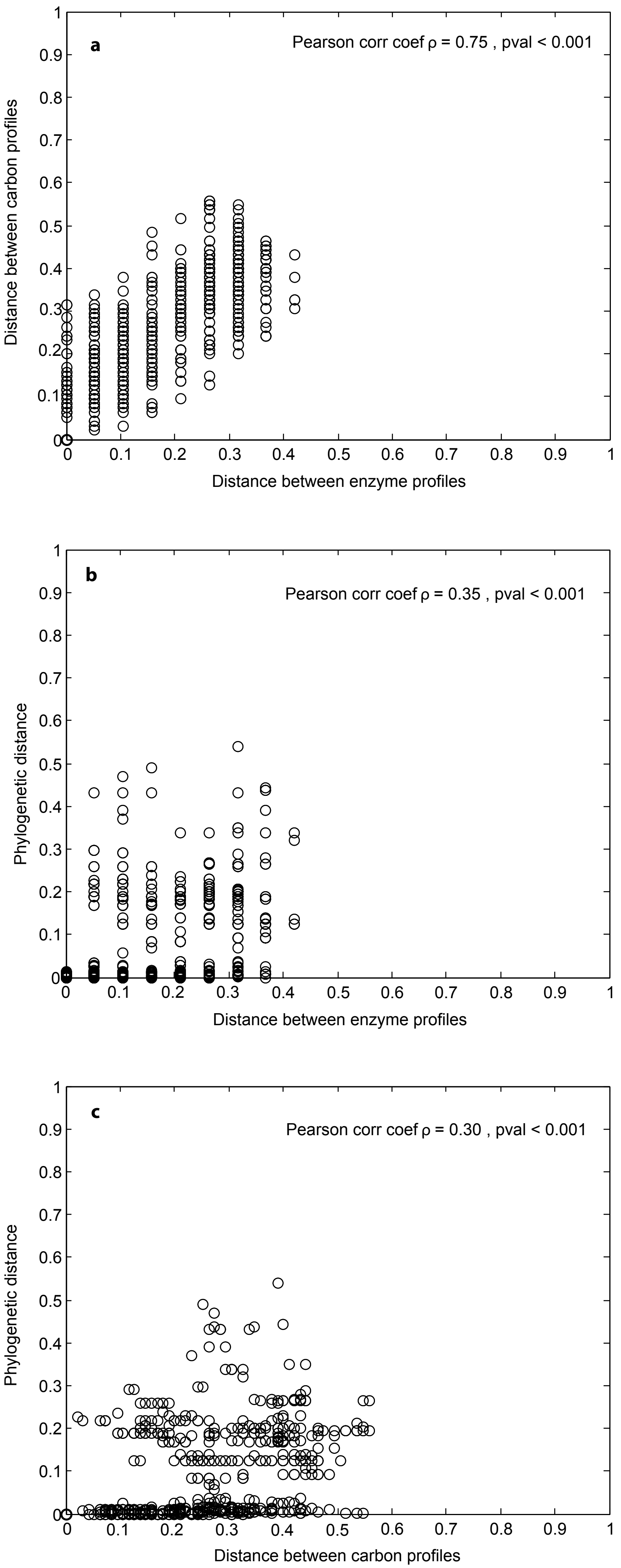
a
Pearson corr coef ρ = 0.75 , pval < 0.001
Distance between carbon profiles
Distance between enzyme profiles
b
Pearson corr coef ρ = 0.35 , pval < 0.001
Phylogenetic distance
Distance between enzyme profiles
c
Pearson corr coef ρ = 0.30 , pval < 0.001
Phylogenetic distance
Distance between carbon profiles

References for supporting information

S1. Zwickl DJ (2006) Genetic algorithm approaches for the phylogenetic analysis of large biological sequence datasets under the maximum likelihood criterion: The University of Texas at Austin.

S2. Posada D (2008) jModelTest: Phylogenetic model averaging. Mol Biol Evol 25: 1253-1256.

S3. Reddy GSN, Matsumoto GI, Schumann P, Stackebrandt E, Shivaji S (2004) Psychrophilic pseudomonads from Antarctica: *Pseudomonas antarctica* sp nov., *Pseudomonas meridiana* sp nov and *Pseudomonas proteolytica* sp nov. Int J Syst Evol Micr 54: 713-719.

S4. Salwan R, Gulati A, Kasana RC (2010) Phylogenetic diversity of alkaline protease-producing psychrotrophic bacteria from glacier and cold environments of Lahaul and Spiti, India. J Basic Microb 50: 150-159.

S5. Christensen AB, Riedel K, Eberl L, Flodgaard LR, Molin S, et al. (2003) Quorum-sensing-directed protein expression in *Serratia proteamaculans* B5a. Microbiol-SGM 149: 471-483.

S6. Geiger A, Fardeau ML, Falsen E, Ollivier B, Cuny G (2010) *Serratia glossinae* sp nov., isolated from the midgut of the tsetse fly *Glossina palpalis gambiensis*. Int J Syst Evol Micr 60: 1261-1265.

S7. Preston-Mafham J, Boddy L, Randerson PF (2002) Analysis of microbial community functional diversity using sole-carbon-source utilisation profiles - a critique. FEMS Microbiol Ecol 42: 1 - 14.
